# Carotenoid Lutein Selectively Inhibits Breast Cancer Cell Growth and Potentiates the Effect of Chemotherapeutic Agents through ROS-Mediated Mechanisms

**DOI:** 10.3390/molecules23040905

**Published:** 2018-04-14

**Authors:** Xiaoming Gong, Joshua R. Smith, Haley M. Swanson, Lewis P. Rubin

**Affiliations:** 1 Department of Pediatrics, Paul L. Foster School of Medicine, Texas Tech University Health Sciences Center, El Paso, TX 79905, USA; lewis.rubin@ttuhsc.edu; 2 Paul L. Foster School of Medicine, Texas Tech University Health Sciences Center, El Paso, TX 79905, USA; J_smith898@yahoo.com (J.R.S.); haley.swanson@ttuhsc.edu (H.M.S.); 3 Department of Biomedical Sciences, Paul L. Foster School of Medicine, Texas Tech University Health Sciences Center, El Paso, TX 79905, USA

**Keywords:** carotenoids, lutein, breast cancer cells, growth inhibition, ROS, chemotherapeutic agents

## Abstract

Increasing evidence suggests that dietary carotenoids may reduce the risk of breast cancer. However, anti-breast cancer effects of carotenoids have been controversial, albeit understudied. Here, we investigated the effects of specific carotenoids on a wide range of breast cancer cell lines, and found that among several carotenoids (including β-carotene, lutein, and astaxanthin), lutein significantly inhibits breast cancer cell growth by inducing cell-cycle arrest and caspase-independent cell death, but it has little effect on the growth of primary mammary epithelial cells (PmECs). Moreover, lutein-mediated growth inhibition of breast cancer cells is quantitatively similar to that induced by chemotherapeutic taxanes, paclitaxel and docetaxel, and exposure to lutein plus taxanes additively inhibits breast cancer cell growth. Analysis of mechanisms showed that lutein treatment significantly increases the intracellular reactive oxygen species (ROS) production in triple-negative breast cancer (TNBC) cells, but not in normal PmECs. Lutein-induced growth inhibition is also attenuated by the radical oxygen scavenger *N*-acetyl cysteine, suggesting a role for ROS generation in the growth inhibitory effect of lutein on TNBC cells. Additionally, we found that the p53 signaling pathway is activated and HSP60 levels are increased by lutein treatment, which may contribute partly to the induction of growth inhibition in TNBC cells. Our findings show that lutein promotes growth inhibition of breast cancer cells through increased cell type-specific ROS generation and alternation of several signaling pathways. Dietary lutein supplementation may be a promising alternative and/or adjunct therapeutic candidate against breast cancer.

## 1. Introduction

Breast cancer remains the second leading cause of cancer-related death and the most frequently diagnosed cancer in American women between the ages of 20 and 59, with over 233,000 new cases and 40,000 deaths annually [1]. Globally, it is the leading cause of cancer deaths in women. Metastatic breast cancer has had a 5-year relative survival rate under 25% [1]. It is a multistep disease involving genetic and environmental factors [2]. In a more granular perspective, breast cancer is a heterogeneous disease which is classifiable into several molecular subtypes, including luminal A, luminal B, human epidermal growth factor (EGF) receptor 2 (HER2) overexpressing, and basal-like. Expression of the estrogen receptor (ER), progesterone receptor (PR), and HER2 are useful markers for identifying these four major subgroups: luminal A: ER^+^ and/or PR^+^/HER2^−^; luminal B: ER^+^ and/or PR^+^/HER2^+^; HER2 overexpressing: ER^−^/HER2^+^; and basal-like: ER/PR^−^/HER^−^. The basal-like subtype, ‘triple negative’ for ER, PR, and HER2, is resistant to available receptor-targeted therapies. Triple-negative breast cancers (TNBC) account for approximately 15 to 20% of all diagnosed breast cancer cases, and are more prevalent in younger women (<40 years of age) and women of African-American or Hispanic origin [2,3]. Currently, aside from systemic chemotherapy, there is no defined standard treatment strategy for prevention of re-occurrence for TNBC. In addition, the lack of selectivity of many cancer chemotherapeutics severely limits current treatment regimes, especially in triple negative tumors and chemo-resistance often emerges in patients having locally advanced or metastatic disease. Consequently, there is an urgent need for identifying new selective and/or nontoxic agents that exhibit anti-cancer activities, and that can lower co-administered effective doses of current non-selective agents.

Carotenoids are primarily plant-derived lipophilic pigments with polyisoprenoid structures. Epidemiological studies indicate that women who have higher levels of circulating dietary carotenoids, such as α-carotene, β-carotene, lycopene, and lutein/zeaxanthin, have significantly lower risk of breast cancer, especially the ER^−^ subtype [4,5]. A pooled analysis of 18 prospective cohort studies further supports an inverse association between intakes of carotenoids and breast cancer risk [6]. However, findings from several other epidemiological studies on the relationship between total carotenoids and the risk of breast cancer have been inconclusive. In sum, some case-control studies and cohort studies have noted an inverse association of breast cancer with specific carotenoids [7,8], while others have not [9]. Moreover, which carotenoids may be active in vitro and in vivo against breast cancer, the respective intracellular mechanisms, and possible interactions with chemotherapy have either not been thoroughly examined or are incompletely understood.

The anti-cancer properties of specific carotenoids may result variously from their antioxidant properties, interactions with cellular (including growth control) signaling cascades, and/or altering gene expression. Certain carotenes, especially β-carotene, are also precursors for vitamin A (retinol) formation. All carotenoids, to different degrees, can act as antioxidants by virtue of free radical quenching properties [10]. However, studies conducted in animal models and human subjects have been less than conclusive regarding direct carotenoid antioxidant effects in vivo [11]. Moreover, clinical trials of high dose of β-carotene for preventing lung cancer failed to show benefit and, rather, produced deleterious effects [12]. A meta-analysis confirmed that a significant increase in mortality was associated with the vitamin A/β-carotene/vitamin E supplement [13].

Importantly, in contrast to β-carotene, the carotenoid lutein has no known toxicities, even in individuals who have ingested it in pharmacologic levels [14]. Previous investigations have revealed that lutein possesses a range of biological properties including anti-inflammatory, anti-oxidant and anti-cancer actions [15], and has growth inhibitory and cytotoxic effects in several cancer cell lines and animal models [15,16]. Lutein inhibits growth of rat prostate carcinoma cells (AT3 cells) and human prostate cancer cells (PC3) [17], alters mouse mammary tumor development [18], and induces apoptosis in transformed but not in normal human mammary cells [19].

In the present study, we investigated the potential effectiveness of an array of carotenoids applied in a nutritional/physiological concentration range against human breast cancer cells and the mechanisms underlying their activities. Our data show that lutein selectively inhibits the growth of all human breast cancer cell lines tested—regardless of receptor phenotype—and enhances the cytotoxic effects of chemotherapeutic taxanes. Importantly, we demonstrate that the pro-apoptotic effect of lutein in triple-negative breast cancer cells is mediated by an increase in intracellular ROS, activation of p53 signaling, and upregulation of cellular HSP60.

## 2. Results

### 2.1. Lutein Selectively Inhibits the Growth of Human Breast Cancer Cells

**Figure 1:**
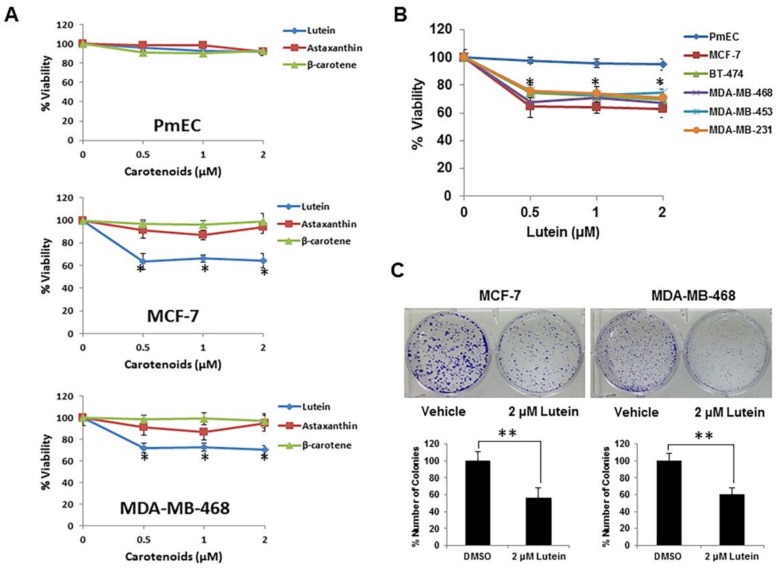
Effect of lutein on growth in human breast cancer cells and normal human breast epithelial cells. (**A**) Lutein treatment inhibits cell growth in human breast cancer cells but not in normal mammary epithelial cells. PmEC and breast cancer MCF-7 and MDA-MB-468 cell lines were grown in 96-well plates and treated with lutein (0.5–2.0 μM) or Dimethyl Sulfoxide (DMSO) for 48 h. Cell viability was measured by MTT assay. The experiments were performed in triplicate. Data are presented as mean ± S.D and are representative of three independent experiments; * *p* < 0.05. (**B**) Inhibition of cell proliferation by lutein in representative breast cancer cell lines and PmEC. Data are presented as means ± S.D. * *p* < 0.05, compared with control cells. (**C**) Colony formation assays were performed in MCF-7 and MDA-MB-468 cells. Breast cancer cells were seeded in 6-well plates at a density of 5 × 10^3^ cells/well, and treated with lutein (2.0 μM) or DMSO (vehicle control) for 48 h. Media was changed after 48 h of incubation. Colonies were monitored for a period of 2 weeks. The data shown are from a representative experiment, repeated three times with similar results. Data are presented as means ± S.D. ** *p* < 0.01, compared with untreated cultures.

Carotenoids may exhibit anti-cancer activity in a variety of cancer cell types [20]. To determine the effects of specific carotenoids on human breast cancer cells, we initially investigated the anti-proliferative effect of three carotenoids (lutein, β-carotene, astaxanthin) on the growth of human breast cancer cell lines (MCF-7 [ER/PR^+^HER2^−^] and MDA-MB-468 [triple-negative]), and normal primary human mammary epithelial cells (PmECs). Astaxanthin, a dietary carotenoid consumed in fish, has anti-inflammatory and suspected anti-tumor activities. Cells were treated with physiological concentration of carotenoids (0.5 to 2.0 μM) for 48 h and cell growth/viability was assayed. As shown in Figure 1A, lutein, but neither β-carotene nor astaxanthin, significantly inhibited the cell viability of both MCF-7 and MDA-MB-468 cells. PmECs exposed to this carotenoid concentration range showed no changes in cell growth/viability. Consequently, we focused on the effects of lutein in normal breast epithelial cells and breast cancer cells.

We examined the effect of lutein on a broader panel of human breast cancer cells, including BT-474 (ER/PR^+^HER2^+^), MDA-MB-453 (triple-negative), and MDA-MB-231 (triple-negative) cell lines, and found that all showed similar lutein-mediated growth inhibition profiles (Figure 1B). To study the effects of lutein on longer-term proliferation, MCF-7 and MDA-MB-468 cells were treated with lutein in colony formation assays. As shown in Figure 1C, lutein treatment significantly reduced colony numbers and decreased the size of colonies formed.

### 2.2. Lutein Induces Cell Cycle Arrest in Human Breast Cancer Cells

**Figure 2:**
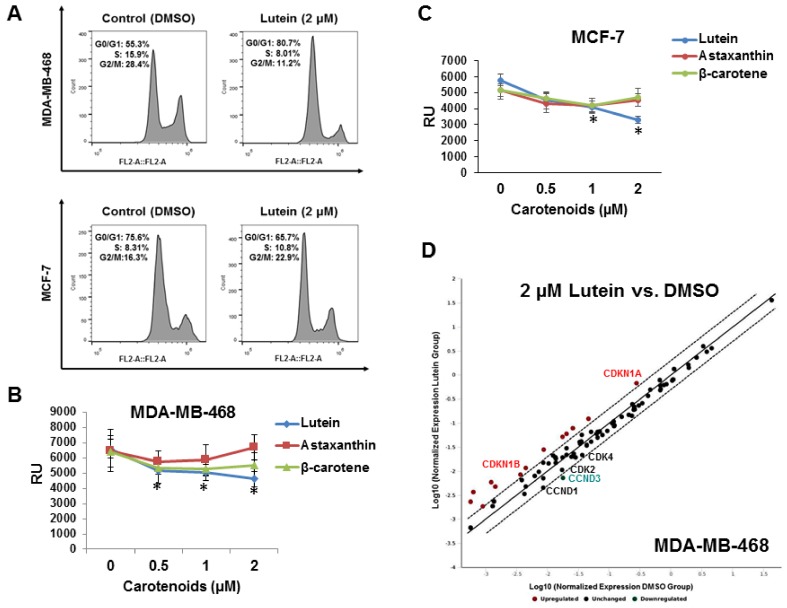
Effect of lutein on cell cycle distribution in triple-negative MDA-MB-468 cells. (**A**) Lutein induced G1 cell cycle arrest in MDA-MB-468 cells and G2 arrest in MCF-7 cells. MDA-MB-468 and MCF-7 cells were treated with 2 μM lutein for 48 h. 0.1% DMSO vehicle was used as control. The distribution of cell cycle phases was assessed by flow cytometry. (**B**) and (**C**) EdU proliferation assays were performed in MDA-MB-468 and MCF-7 cells. Breast cancer cells were grown in 96-well plates and treated with carotenoids (lutein, β-carotene, astaxanthin) at 0.5–2.0 μM or DMSO for 48 h. Data shown represent mean ± S.D. (*n* = 6); * *p* < 0.05. (**D**) The scatter plot compares the normalized expression of every gene on the array between the two groups (lutein vs. DMSO) by plotting them against one another to quickly visualize large gene expression changes. The central line indicates unchanged gene expression. The dotted lines indicate the 2-fold regulation threshold.

To investigate mechanism(s) underlying lutein’s inhibitory activity on breast cancer cells, we first examined the effects of lutein on cell cycle progression. MCF-7 and MDA-MB-468 cells were treated with 2.0 μM lutein or vehicle for 48 h, and then subjected to cell cycle analysis by flow cytometry. Lutein treatment significantly inhibited cell cycle progression in both MDA-MB-468 and MCF-7 cells, resulting in an increased population of cells in G1 phase and a reduction in G2 phase in MDA-MB-468 cells, as well as a decreased cell population in G1 phase and an increase in G2 phase in MCF-7 cells (Figure 2A). At 48 h, the percentage of cells in G1 had increased from 55.3% in control cells to 80.7% in lutein-treated MDA-MB-468 cells. Similarly, the percentage of cells in S phase decreased to 8.0% from 15.8% in control cultures; G2 phase cells decreased to 11.2% from 28.4% in the control (Figure 2A *upper panel*). DNA incorporation assays using the modified thymidine analog EdU confirmed the observed decline in S phase in lutein-, but not β-carotene or astaxanthin-treated MDA-MB-468 cells (Figure 2B). Similar effects of three carotenoids on MCF-7 cells (Figure 2C) were also observed. To further investigate the underlying mechanisms responsible for lutein-induced G1 arrest in MDA-MB-468 cells, we analyzed cell-cycle related gene expression profiles using targeted cell cycle regulatory gene expression PCR arrays. As shown in Figure 2D, lutein-treated MDA-MB-468 cells exhibited significant up-regulation of anaphase promoting complex subunit 2 (ANAPC2), aurora kinase B (AURKB), cyclin F (CCNF), cell division cycle 25 homolog A (CDC25A), cyclin-dependent kinase 6 (CDK6), CDKN1A (p21, Cip1), CDKN1B (p27, Kip1), E2F transcription factors 1 and 4 (E2F1, E2F4), minichromosome maintenance complex component 2, and 5 (MCM2, and 5), checkpoint controller RAD9A, SERTAD1 and transcription factor Dp-1 (TFDP1), and down-regulation of CCND1 (Cyclin D1), CCND3 (Cyclin D3), CDK2 and CDK4.

### 2.3. Lutein Induces Minimal Apoptotic Cell Death in Breast Cancer Cells

**Figure 3:**
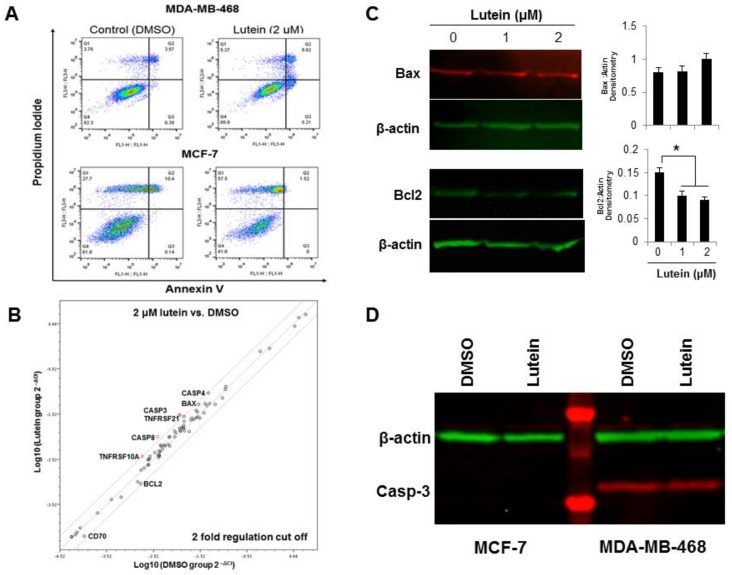
Pro-apoptotic effects of lutein on breast cancer cells. (**A**) MDA-MB-468 and MCF-7 cells were exposed to 2.0 μM lutein for 24 h. DMSO was used as vehicle control. Cells were processed by flow cytometry using Annexin V/PI staining. The percentage of Annexin V-positive cells indicates the fraction of cells undergoing apoptosis induction. (**B**) Relative expression of 84 apoptosis-related genes comparing lutein-treated MDA-MB-468 cells and DMSO vehicle control. The figure depicts a log transformation plot of the relative expression level of each gene (2^−ΔCt^) between untreated control (x-axis) and lutein treatment (y-axis). The gray lines indicate a 2-fold change in gene expression. (**C**) Changes in levels of apoptosis-related proteins by lutein in MDA-MB-468 cells. Cells were treated with 1.0 and 2.0 μM lutein for 24 h and cell lysates were prepared and subjected to Western blotting for Bax and Bcl-2. The histogram in Figure 3C indicates the average of adjusted Bax and Bcl2 levels (mean ± SD, *n* = 3). * *p* < 0.05. (**D**) Western blot analysis of caspase-3 cleavage in MDA-MB-468 and MCF-7 cells. Cells were treated with 2.0 μM lutein for 24 h and cell lysates were prepared and subjected to Western blot for caspase-3.

To investigate if lutein-mediated reduction in cell proliferation results from apoptosis, annexin V-FITC/propidium iodide (PI) double staining was used, in order to determine cell death quantitatively in lutein-treated vs untreated MDA-MB-468 and MCF-7 cells. Treatment of MDA-MB-468 (Figure 3A, upper panel) or MCF-7 cells (Figure 3A, lower panel) with lutein (2.0 μM for 24 h) did not significantly alter the early stage apoptotic (annexin V^+^/PI^−^) population (5.21%) in MDA-MB-468 cells. An increased, but still minor (<10%) late-stage apoptotic/necrotic (annexin V^+^/PI^+^) cell fraction was also observed in MDA-MB-468 cells, but not in MCF-7 cells. To investigate if lutein induces cell death by triggering the mitochondrial apoptotic pathway, we examined the expression of a panel of apoptosis-related genes using gene expression profiling RT-PCR arrays. As shown in Figure 3B, lutein-treated MDA-MB-468 cells exhibited increased expression in seven pro-apoptotic genes (GADD45A, Bax, CASP3/4/8, TNFRSF10A and TNFRSF21) decreased expressions in one pro-apoptotic gene (CD70), and one anti-apoptotic gene (Bcl-2). Consistent with the gene expression profiling data, lutein-treated MDA-MB-468 cells showed a slight increase in the protein level of Bax and a larger decrease in Bcl-2, which results in a two-fold increase in the pro-apoptotic/anti-apoptotic (Bax:Bcl-2) ratio (Figure 3C). Caspase-3 activation is a hallmark of an apoptotic pathway. We examined the levels of cleaved caspase-3 in lutein-treated MDA-MB-468 and MCF-7 cells. Despite increased caspase-3 gene expression in MDA-MB-468 cells, we only detected minimal caspase-3 cleavage in lutein-treated MDA-MB-468 cells (Figure 3D), and as well as caspase 3 deficiency in MCF-7 cells.

### 2.4. Lutein Selectively Increases Intracellular ROS Production in Human Breast Cancer Cells

**Figure 4:**
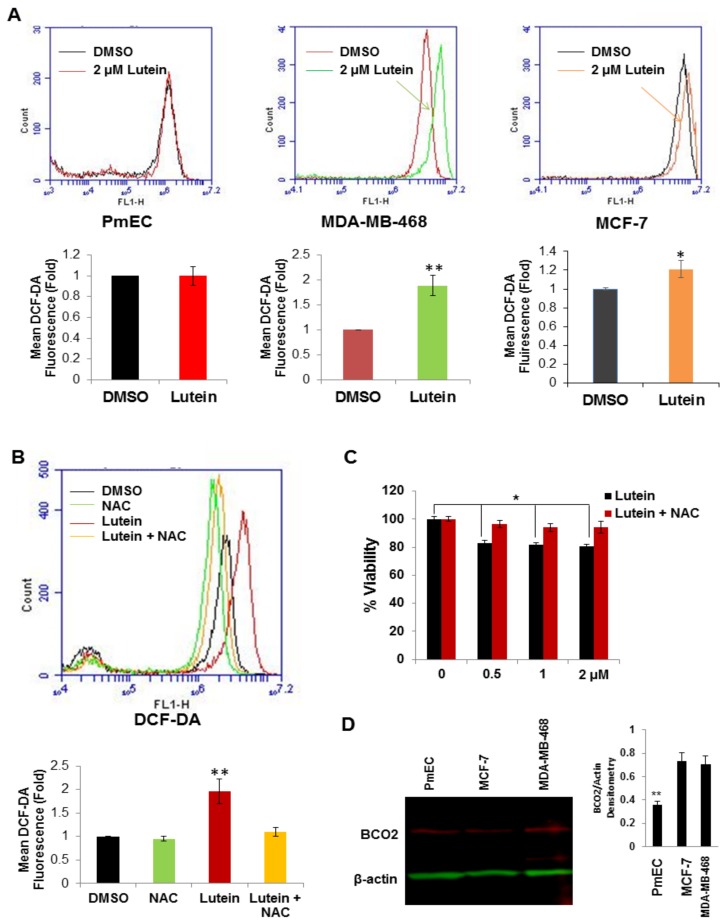
Effect of lutein on intracellular ROS production in human normal and breast cancer cells. (**A**) PmECs, MDA-MB-468 and MCF-7 cells were treated with 2.0 μM lutein for 3 h and intracellular ROS levels were measured by flow cytometry. Data are presented as means ± S.D. and are representative of three independent experiments. * *p* < 0.05, ** *p* < 0.001, compared with control group. (**B**) MDA-MB-468 cells were pretreated with or without 3.0 mM NAC for 1 h, and followed by 2.0 μM lutein for 3 h. Lutein induced an increase in ROS levels which is abolished by NAC. Data are presented as means ± S.D. and are representative of three independent experiments. ** *p* < 0.01, compared with the indicated group. (**C**) NAC blocked the growth inhibitory effect of lutein on breast cancer cells. MDA-MB-468 cells were grown in 96-well plates, and pre-treated with or without 3.0 mM NAC for 1 h, followed by lutein (0.5–2.0 μM) or DMSO (control) for 48 h. Cell viability was measured by MTT assay. The experiments were performed in triplicate. Data are presented as mean ± S.D and are representative of three independent experiments; * *p* < 0.05). (**D**) Effect of lutein on expression of BCO2. PmEC, MCF-7 and MDA-MB-468 cells were gown in 6-well plate for 48 h. Cell lysates were prepared and subjected to Western blotting for BCO2. The histogram in Figure 4D indicates the average of adjusted BCO2 levels (mean ± SD, *n* = 3), ** *p* < 0.01.

Although carotenoids (including lutein) are often classified as anti-oxidants, in biological systems carotenoids may exhibit either anti- or pro-oxidant activities [21]. To determine if lutein affects intracellular ROS levels in breast cancer cells, we used the redox-sensitive fluorescent probe (carboxy-H_2_DCF-DA) to monitor intracellular ROS accumulation in the presence and absence of lutein exposure. Breast cancer cells treated with lutein (2.0 μM for 3 h) significantly increased ROS levels (1.9-fold) in MDA-MB-468 cells, compared to untreated (DMSO vehicle) cells (Figure 4A, *middle panel*), and slightly elevated ROS levels in MCF-7 cells (Figure 4A, *right panel*). In contrast to the results in MDA-MB-468 and MCF-7 cells, similar exposures to lutein did not significantly increase in ROS levels in normal PmECs (Figure 4A, *left panel*).

To assess the functional importance of ROS induction in lutein-treated triple-negative MDA-MB-468 cells, we examined lutein-inducible cellular ROS generation in cells pretreated with the ROS scavenger, *N*-acetyl cysteine (NAC). MDA-MB-468 cells were pretreated with or without NAC (3.0 mM for 1 h), and then exposed to lutein for an additional 3 h. As anticipated, pretreatment of cells with NAC caused a significant drop in ROS levels in lutein-treated MDA-MB-468 cells (Figure 4B). Moreover, blocking ROS generation by NAC also abolished the growth inhibitory effect of lutein (Figure 4C). These results suggest that the selective lutein-mediated growth inhibition of breast cancer cells is mediated by ROS induction. Previous investigations have suggested that carotenoids (including lutein/zeaxanthin) induce intracellular ROS production in cells when the carotenoid (lutein) metabolic enzyme β-carotene 9’,10’-oxygenase (BCO2) is absent [22]. To determine if lutein-induced ROS production in breast cancer cells is dependent on the expression of BCO2, we analyzed BCO2 protein expression in normal and breast cancer cell lines by western blot. As shown in Figure 4D, the level of BCO2 in normal breast epithelial cells is actually slightly lower than that in the lutein sensitive breast cancer lines, implying that lutein-selective induction of ROS in breast cancer cells may not BCO2-dependent.

### 2.5. Lutein Enhances the Cytotoxic Effects of Chemotherapeutic Taxanes in Human Breast Cancer Cells

**Figure 5:**
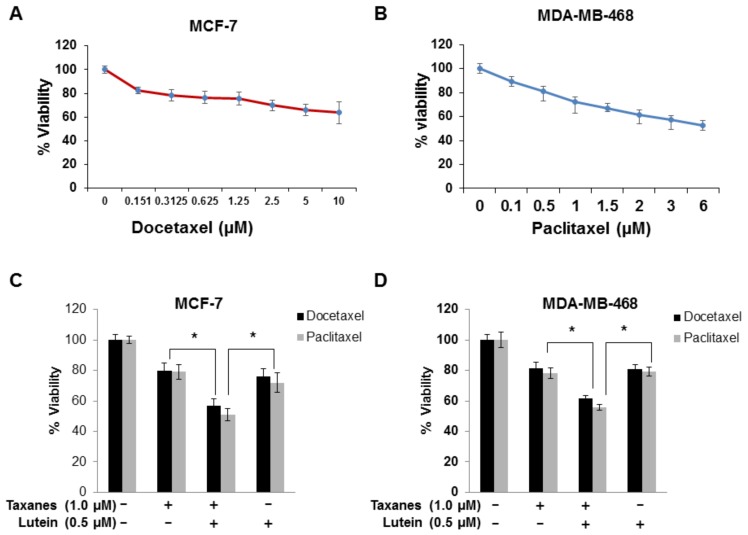
Enhanced effects of lutein and chemotherapeutic taxanes in human breast cancer cells. (**A**) MCF-7 cells were treated with docetaxel for 24 h in a dose-dependent manner and MTT assays were used to assess cytotoxic and growth effects of exposure to docetaxel. (**B**) MDA-MB-468 cells were treated with paclitaxel for 24 h in a dose-dependent manner, and cell viability was measured using MTT assays. The experiments were performed in triplicate. Data are presented as mean ± S.D and are representative of three independent experiments. (**C**) MCF-7 cells were treated with taxanes alone (1.0 µM), lutein alone (0.5 µM) and lutein in combination with a taxane. (**D**) MDA-MB-468 cells were treated with taxanes alone (1.0 µM), lutein alone (0.5 µM) and lutein in combination with a taxane. Experiments were performed in triplicate. Data are presented as means ± S.D and are representative of three independent experiments; * *p* < 0.05.

Taxanes, a major class of breast cancer chemotherapeutic agents, are microtubule disruptors, i.e., mitotic inhibitors. Our observation that caspase-independent cell death induced by lutein through a ROS-mediated mechanism in breast cancer cells raised the possibility that this ‘nutriceutical’ might be a useful adjunctive therapeutic option with chemotherapy. To investigate this possibility, we first assessed the sensitivity of MCF-7 and MDA-MB-468 cells to increasing concentrations of paclitaxel or docetaxel, comparable to those used in breast cancer treatment. MTT assays indicated a dose-dependent decrease in cell proliferation in docetaxel-treated MCF-7 cells (Figure 5A) and paclitaxel-treated MDA-MB-468 cells (Figure 5B). Taxane-mediated growth inhibition of breast cancer cells with a concentration of 1.0 µM was quantitatively similar to that induced by a similar concentration of lutein. To determine if lutein can sensitize breast cancer cells to taxane-mediated cell growth inhibition, we examined the effect of combinations of lutein and either paclitaxel or docetaxel on cell viability using MCF-7 and MDA-MB-468 cells. Following 24 h treatment, lutein (0.5 µM) or taxane (1.0 µM) alone reduced MCF-7 and MDA-MB-468 cell viability by 20% and 25%, respectively. The combination of 0.5 µM lutein and 1.0 µM paclitaxel or docetaxel resulted in 50% and 40% growth inhibition of MCF-7 (Figure 5C) and MDA-MB-468 (Figure 5D) cells respectively. These data indicate that lutein and taxanes together additively inhibit breast cancer cell growth, supporting the hypothesis that lutein may potentiate the effects of chemotherapeutic taxanes.

### 2.6. Lutein Activates the p53 Signaling Pathway and Upregulates HSP60 in MDA-MB-468 Cells

**Figure 6:**
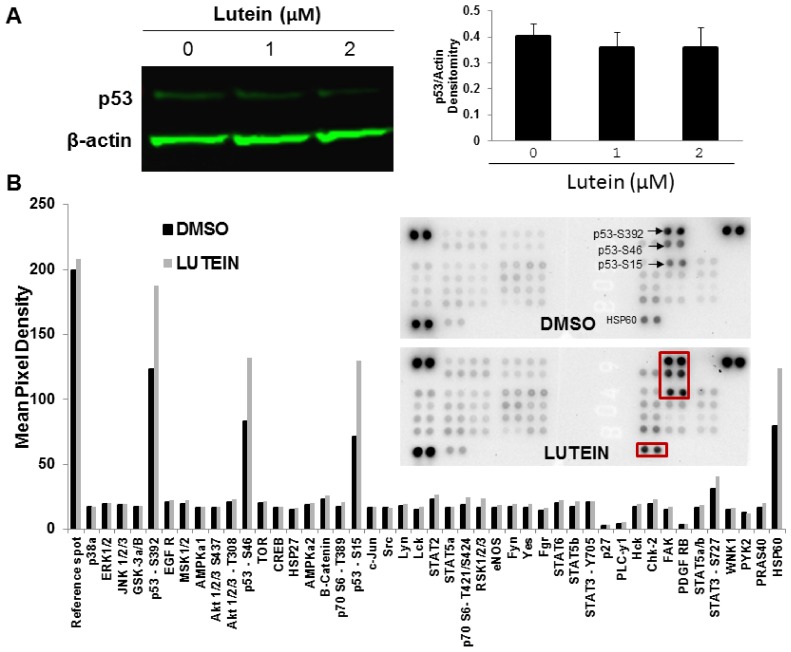
Effect of lutein on phosphorylated signaling proteins in triple-negative breast cancer cells. (**A**) Left panel: Western blot analysis of p53 in MDA-MB-468 cells. Cells were treated with 1.0, or 2.0 μM lutein or DMSO vehicle control for 24 h. Cell lysates were prepared and subjected to Western blotting for p53. Right panel: the histogram in Figure 6A indicates the average of adjusted p53 levels (mean ± SD, *n* = 3). (**B**) Analysis of a proteome profiler for human phospho-kinases using breast cancer MDA-MB-468 cell lysates from lutein-treated and DMSO vehicle control. Data represent the densitometric analysis of phosphorylated signaling proteins in MDA-MB-468 cells treated with lutein or control. The insets are the representative proteome profiler from the Human Phospho-Kinase Array using MDA-MB-468 cell lysates treated with 2.0 μM lutein or DMSO control for 12 h.

Increased cancer cell ROS production that is associated with targeted cancer killing [23] also sustains elevated cancer proliferative demands, in effect, driving tumor cells toward a redox stress threshold and increasing tumor susceptibility to ROS-modulating agents. Many anti-cancer compounds induce ROS generation and activate related cell signaling pathways [24]. To determine if lutein activates pathways related to mitochondrial oxidation and apoptosis, we examined targeted protein phosphorylation in lutein-treated vs. vehicle-treated MDA-MB-468 cells. MDA-MB-468 cells were exposed to 2.0 μM lutein for 12 h and protein phosphorylation was examined using Western blot and a human phospho-kinase array. Lutein treatment did not alter the level of total p53 protein in MDA-MB-468 cells (Figure 6A), but, as shown in Figure 6B, significantly increased levels of phosphorylated p53. Cellular HSP60 also was robustly increased in response to lutein treatment.

## 3. Discussion

Based on a range of epidemiologic and experimental data, specific carotenoids have been implicated as potential anti-cancer agents in several tumor types [15,20]. In this study, we evaluated the growth inhibitory effects of several carotenoids (β-carotene, lutein, and astaxanthin) in normal and breast cancer cells. Although β-carotene, lutein, and astaxanthin resemble one another structurally, they have distinct biological actions. We demonstrate that human breast cancer cell lines, but not normal human primary mammary epithelial cells, are highly sensitive to growth inhibition by exposure to the carotenoid lutein, while β-carotene and astaxanthin, two other carotenoids having biological activity in other systems, had no effects. Lutein also induced cell cycle arrest and increased apoptosis in triple negative breast cancer cells. Importantly, intracellular ROS levels were induced by lutein in TNBC cells, but not in primary mammary epithelial cells. This increased ROS production, in turn, appears to mediate lutein-mediated growth inhibition in breast cancer cells. These findings suggest mechanisms that may underlie lutein’s selective cytotoxic effects in breast tumors.

Apoptosis plays a pivotal role in carcinogenesis, including cancer initiation, progression, and metastasis [25]. Apoptosis can be induced through either extrinsic or intrinsic pathways. The extrinsic pathway is triggered by death receptors (Fas, TNFR, DR5) in response to ligand binding, leading to caspase-8 activation [26]. A key event in the intrinsic pathway is permeabilization of the mitochondrial outer membrane in response to various stimuli, a process that is regulated by cytoplasmic proteins including Bcl-2 family members [27]. Bcl-2 is a thiol-containing protein that prevents apoptosis through the negative regulation of Bax and Bak mitochondrial translocation [28]. The Bax/Bcl-2 ratio, as a candidate prognostic biomarker in breast cancer, indicates the degree of mitochondrial outer membrane permeabilization and, hence, cell entry for the execution phase of the apoptotic program. The present study shows that a decrease in Bcl-2 expression is accompanied by concomitant increases in Bax protein expression in response to lutein (Figure 3B,C). Although caspase-dependent apoptosis appears to be the major form of controlled cell death in cancer cells, lutein did not appear to activate caspase-3 cleavage in breast cancer cells, suggesting lutein might induce cell apoptotic cell death through caspase-independent mitochondrial pathway.

Over the past decades, carotenoid research on cancers has focused on prevention, prompted by the purported carotenoid functions as antioxidants. Indeed, cell culture, animal, and epidemiologic studies have suggested that various carotenoids may be protective against tumorigenesis [20]. However, two large clinic trials that utilized high dose β-carotene administration showed an increased risk in lung cancer among smokers, implying potential pro-oxidant activities of β-carotene [29]. In fact, some carotenoids, including β-carotene, can act either as anti- or pro-oxidants depending on concentration and the targeted cells [21,29,30]. With regard to cellular oxidant stress, it has been reported that lutein can inhibit methotrexate-induced apoptosis in IEC-6 cells by blocking ROS generation [31]. Conversely, lutein can induce intracellular ROS production in macrophages [32] and mitochondrial-mediated ROS generation to trigger apoptosis in HepG2 (human hepatic carcinoma) cells [33]. Lutein also has been shown to exert significant inhibitory effects on deoxynivalenol-induced apoptosis in HT-29 cells, possibly due to its anti-oxidant and anti-inflammatory activities [34].

In this study, we show that lutein has potent anti-proliferative and potentially cytotoxic effects in breast cancer cells, but has little effect on normal human breast epithelial cell growth or viability. Lutein also significantly increases ROS production in triple-negative breast cancer (MDA-MB-468) cells, but not in primary mammary epithelial cells. The ROS scavenger NAC significantly attenuated lutein-mediated cell death in lutein-treated triple-negative breast cancer cells. Cancer cells usually exhibit excessive ROS production compared to normal cells, related to aberrant cell metabolism and continuous cell division, and are less tolerant to further oxidative (ROS) insult. Another natural product, piperlongumine, has been reported selectively to increase ROS levels and apoptotic cell death in cancer cells, but again, not in normal cells [35]. Vitamin C also selectively kills KRAS and BRAF mutant cells by inducing oxidative stress [36]. Cell-based and animal studies have indicated certain dietary carotenoids (e.g., lutein and, its geometric isomer zeaxanthin) can impair hepatic mitochondrial respiration, increase mitochondrial ROS generation, and induce apoptotic cell death [22]. These effects are proposed to result from interference with mitochondrial electron transport and are dependent on the mitochondrial-localized carotenoid metabolic enzyme, β-carotene 9’,10’-oxygenase (BCO2) [33]. BCO2-mediated carotenoid metabolism protects mitochondria from carotenoid-induced dysfunction. Not surprisingly, the *bco2* gene is expressed in human epithelial cells and most human tissues [37]. Conversely, bco2 gene expression is decreased in several cancers. We previously determined that BCO2 is highly expressed in normal prostatic epithelial cells, but its expression is decreased and even lost in many prostate cancer cell lines and tissues [38]. However, in contrast to prostate cancer, we now show that BCO2 expression in breast cancer cells is even greater than that in normal breast epithelial cells (Figure 4D), which suggests that the observed lutein-mediated increase in ROS generation may not be BCO2-dependent in breast cancer. The role of BCO2 accumulation in breast cancer cells has not previously been explored. Of note, unlike β-carotene, which is localized in the cytoplasm of cells, lutein/zeaxanthin and their oxidized metabolites are enriched in the inner membranes of mitochondria of hepatic cells [39]. In summary, further investigation is needed to determine mechanisms underlying lutein-induced ROS generation in breast cancer.

Activation of caspases is a common means by which cancer cells undergo type-1 programmed cell death [40]. Nevertheless, many tumor cells develop resistance by becoming deficient in this apoptotic pathway, thereby, averting death [40]. Consequently, exploiting a combination of various mechanisms of cell death may be a viable approach to induce synergistic cancer cell killing. We present the novel finding that lutein potentiates the lethality of taxanes (Figure 5C,D). Our results suggest this interaction might act through reciprocal induction of caspase-dependent and caspase-independent pathways. Although the molecular mechanisms of the interaction of lutein and taxanes remain to be determined, these findings suggest strategies that target both caspase-dependent and caspase-independent pathways may improve outcomes compared with conventional therapies.

Multiple lines of evidences support the contention that excessive ROS release and DNA damage promote apoptosis by common signaling pathways. The current findings of lutein-inducible cell cycle arrest and DNA damage suggest involvement of p53. The major tumor suppressor protein, p53, is one of the most commonly mutated genes associated with human cancer [41], and is a crucial regulator of cell growth and death. In response to intracellular and extracellular stress, p53 is activated via phosphorylation, and serves as a transcription factor in a range of target genes which, in turn, modulate cellular processes such as DNA repair, cell cycle arrest, and apoptosis [42]. Mounting evidence suggests that p53-dependent apoptosis is mediated by intracellular ROS generation [43]. Many anti-cancer agents (e.g., cisplatin, paclitaxel, docetaxel, anthracyclins, adriamycin, etoposide) are directly or indirectly toxic to cancer cells, in part, by generating ROS leading to apoptotic cell death [24]. Consistent with this mechanism, we demonstrate that lutein selectively increases intracellular ROS production in triple-negative MDA-MB-468 breast cancer cells and induces significant elevations in p53 phosphorylation at serine residues Ser15, Ser46, and Ser392 (Figure 6A,B). Phosphorylation of wild-type p53 at Ser15 plays a major role in the cellular response to DNA damage by reducing interaction between p53 and its negative regulator, the ubiquitin ligase MDM2. Phosphorylation of p53 at Ser392 also appears to be important in growth suppression, DNA binding, and transactivation of wild-type p53 [44]. Of note, an important characteristic of the MDA-MB-468 cell line is the presence of a single p53 allele that harbors a point mutation at codon 273 (p53R273H). Mutations of p53 lead to loss of wild-type activity either by altering a residue that directly interacts with DNA or by mutating a residue that destabilizes or partly unfolds p53 [45,46]. Phosphorylation of mutant p53 at Ser392 inhibits its function as a dominant-negative inhibitor over any remaining wild-type p53 [47]. Our observation that lutein induces p53 phosphorylation in MDA-MB-468 cells might partly explain the observed lutein-mediated growth inhibitory effects.

The observed increase of heat shock protein 60 (HSP60) in lutein-treated breast cancer cells is also novel. Previous studies of HSP60 have focused on total immuno-reactive protein, its subcellular distribution, and HSP60 complex proteins. HSP60, a member of the mitochondrial matrix protein chaperonin family, exists in several multimeric forms and has a wide range of established cellular functions. HSP accumulation (largely in the cytoplasm) may be increased or decreased, depending on cancer type; in some tumors, increased levels of different HSPs have been correlated with a better prognosis [48]. Although most heat shock proteins seem to have pro-survival functions, HSP60 accumulated in the cytosol can be either pro-apoptotic or pro-survival, depending on differential interactions with caspase-3 [49]. Moreover, several observations indicate HSP60 has a pro-apoptotic role in certain cancer cells in vivo [49,50]. Although the distribution and functions of HSP in breast cancer phenotypes are not known in any detail, HSP60 is overexpressed at the mRNA and protein levels during early steps of certain breast tumorigenesis, including breast ductal carcinoma in situ, and may be correlated with tumor growth and progression. In etoposide-stimulated MDA-MB-231, breast cancer cells cytosolic HSP60 levels increased, whereas mitochondrial HSP60 levels and HSP mRNA levels remained constant. Our finding that total cellular HSP60 is increased in lutein-treated breast cancer cells suggests HSP60 may be part of the lutein-induced cellular stress response in these cells. The relationships of HSP60 subcellular (cytosolic vs. mitochondrial) distribution, and protein complexing to an increase in ROS, warrant further investigation.

## 4. Materials and Methods

### 4.1. Antibodies and Reagents

Antibody against BCO2 (Catalog number [Cat#] 14324-1-AP, 1:1000) was acquired from ProteinTech Group (Rosemont, IL, USA). Antibodies against cleaved caspase-3 (Cat# 9662, 1:1000), p53 (Cat# 2524, 1:1000), Bax (Cat#2772, 1:1000) and Bcl-2 (Cat#15071, 1:1000) were obtained from Cell Signaling (Danvers, MA, USA). Antibody to β-actin (Cat# A5441, 1:10,000) was obtained from Sigma Aldrich (St Louis, MO, USA). The 5-(and-6)-chloromethyl-2′, 7′-dichlorodihydrofluorescein diacetate, acetyl ester (CM-H_2_DCF-DA) and *N*-acetyl cystenine (NAC) were purchased from Sigma Aldrich. Lutein was obtained from Kemin Health (Des Moines, IA, USA; FloraGLO Lutein 10% VG TabGrade). Beta-carotene and astaxanthin were obtained from Sigma-Aldrich. Working solutions of carotenoids were freshly prepared with dimethyl sulfoxide (DMSO) and tetrahydrofurlan (THF) in a ratio 2:1 immediately before use. Docetaxel and paclitaxel were obtained from Tocris Bioscience (Minneapolis, MN, USA).

### 4.2. Cell and Cell Culture

Human primary mammary epithelial cells (PmECs, ATCC, PCS-600-010) were purchased from ATCC (American Type Culture Collection, Manassas, VA, USA) and were cultured in Mammary Epithelial Cell Basal Medium + One (ATCC, PCS-600-400). Human breast cancer cell lines (MCF-7, MDA-MB-468) were obtained from Dr. Rajkumar Lakshmanaswamy, Department of Biomedical Sciences, Paul L. Foster School of Medicine, Texas Tech University Health Sciences Center El Paso. Cell lines (BT474, ATCC, CRL-3247; MDA-MB-453, ATCC, HTB-131 and MDA-MB-231, ATCC, HTB-26) were obtained from ATCC. Breast cancer cells were cultured in RPMI medium (GIBCO, Life Technologies, Gaithersburg, MD, USA) supplemented with 10% fetal bovine serum (FBS) (Thermo Fisher Scientific, Waltham, MA, USA), and 100 IU/mL penicillin/streptomycin (Invitrogen, Carlsbad, CA, USA) in a humidified atmosphere of 95% air and 5% CO_2_ at 37 °C.

### 4.3. Cell Proliferation/Viability Assays

Normal PmECs and breast cancer cells were seeded onto 96-well plates at a density of 1 × 10^5^ cells/mL, and left overnight to attach. Culture medium was removed and the cells were treated with serial dilutions of lutein (0.5–2.0 μM) in fresh culture medium with 10% FBS, or cultured in medium with DMSO (in the same concentration as carotenoid samples) as a control. Cell viability was determined after 48 h by MTT assay using CellTiter96® Non-Radioactive Cell Proliferation kits (Promega, Madison, WI, USA), in which the yellow tetrazolium salt [3-(4,5-dimethylthiazol-2-yl)-2,5-diphenyltetrazolium bromide] is reduced by mitochondrial dehydrogenase in viable cells to purple, insoluble crystals of formazan. Cells were incubated for 4 h with MTT solution at 37 °C and formazan crystals were solubilized in a lysing buffer overnight at room temperature (RT). The product was quantified by measurement of absorbance at a 570 nm wavelength with the use of a BioTek Synergy™ H4 Hybrid Multi-Mode Microplate Reader (BioTek Instruments, Winooski, VT, USA). All experiments were performed in triplicate and yielded similar results. Results are representative of an average of three independent experiments. Data are presented as proportional viability (%) by comparing the treated group with the untreated cells, the viability of which was assumed as 100%.

### 4.4. Colony Formation Assay

Breast cancer cells (5 × 10^3^) were seeded per well in 6-well plates and cultured overnight. Cells were then treated with lutein at various concentrations for 48 h. After rinsing with fresh medium, individual cells were allowed to form colonies for the indicated time periods, fixed, and then stained with 0.04% crystal violet. Any colony containing more than 30 cells was counted as one positive colony. The inhibition of colony formation ratio is expressed as the percentage compared to vehicle control.

### 4.5. Cell Cycle Analysis

MDA-MB-468 cells were seeded onto 6-well plates at a density of 3 × 10^5^ cells/mL and left overnight to attach. Culture medium was removed and the cells were exposed to 2.0 μM lutein in fresh culture medium with 10% FBS or cultured in medium with DMSO as a control. After 48 h, treated cells were stained with propidium iodide (PI) using PI/RNase Staining Buffer (BD Biosciences, BD Pharmingen, San Jose, CA, USA), according to the manufacturer’s instructions. Briefly, cells were harvested and centrifuged at 300× *g* for 5 min at RT, and the pellets were fixed in ice-cold 80% ethanol overnight at −20 °C. Following fixation, cells were centrifuged at 300× *g* for 5 min at 4 °C, washed twice in PBS, and then incubated with 0.5 ml PI/RNase solution per 1 × 10^6^ cells for 15 min in darkness at RT. Next, the stained cells were analyzed by flow cytometry using a Accuri C-6 flow cytometer (BD Biosciences) equipped with a 488-nm argon-ion laser, in order to assess the percentage of cells in phases G0/G1, S, and G2/M, based on the amount of PI incorporated into DNA; histograms of DNA distribution were then generated. The PI fluorescence intensity of individual nuclei was determined, and at least 10,000 events were measured within an acquisition rate >60 events/second. Cell cycle analyses were performed with FlowJo software (NIH). All experiments were performed in triplicate and yielded similar results.

### 4.6. EdU Proliferation Assays

MDA-MB-468 and MCF-7 cells were seeded on 96-well plates at a density of 10,000 cells/well (1 × 10^5^/mL) and 5,000 cells/well (5 × 10^4^/mL) respectively, and left overnight to attach. Culture medium was removed and cells were treated with serial dilutions of carotenoids (lutein, β-carotene, astaxanthin at 0, 0.5, 1.0, and 2.0 μM) in fresh culture medium with 10% FBS. Control cells were cultured in medium with the vehicle (DMSO/THF). Cell proliferation was assessed after 48 h with the use of a Cell Proliferation Click-iT EdU Microplate Assay (Life Technologies, Carlsbad, CA, USA), according to the manufacturer’s instructions. All experiments were performed in triplicate and yielded similar results.

### 4.7. Flow Cytometric Assay of Apoptosis

Apoptotic cells were identified by an Annexin V/Dead Cell Apoptosis Kit (Invitrogen) according to the manufacturer’s instructions. Briefly, MDA-MB-468 cells were treated with DMSO (vehicle) or various concentrations of lutein for 24 h. The adherent cells were trypsinized, pelleted, washed in ice-cold PBS, and resuspended in 1× binding buffer. Cells were then stained with FITC–Annexin V and propidium iodide (PI) for 15 min at room temperature in the dark. Annexin V–FITC detects translocation of phosphatidylinositol from the inner to the outer cell membrane during early apoptosis; PI enters cells during late apoptosis or necrosis. Untreated cells were used as control for the double staining. Cells were analyzed immediately after staining using flow cytometry and FlowJo software. For each measurement, at least 20,000 cells were counted.

### 4.8. Quantitative RT–PCR Apoptosis and Cell Cycle Focused Gene Arrays

RT2 Profiler PCR 96-well arrays (SA Biosciences) for the detection of 84 key apoptosis- and cell cycle-related genes respectively, were used quantitatively to determine gene expression. Total RNA was isolated and extracted using an RNA extraction kit (RNeasy Mini Kit, Qiagen, Germantown, MD, USA), according to manufacturer’s protocols cDNA synthesis was performed using a first strand cDNA synthesis kit (SA Biosciences, Frederick, MD, USA), and cDNA amplification was performed using an Amplification Master Mix kit. Samples were prepared with Master Mix and Template Cocktail, and loaded into the PCR array wells and run on a Bio-Rad CX90 thermal cycler using SYBR Green detection. Cycle thresholds were determined for each gene using instrument’ software. Cycle threshold values were transferred into a Data Analysis Template Excel file and analyzed on SA Bioscience’s PCR Array Data Analysis Web Portal.

### 4.9. Determination of Intracellular ROS Production

ROS generation was measured using the oxidation sensitive fluorescent probe CM-H_2_DCF-DA (Life Technologies). Following treatment with the indicated concentrations of lutein in the absence or presence of NAC for the specified time periods, cells were washed with PBS and incubated with 10 µM CM-H_2_DCF-DA for 30 min at 37 °C in the dark. ROS production was analyzed by flow cytometry.

### 4.10. Quantitative Fluorescent Western Blot Analysis

Cells were harvested and lysed in ice-cold M-PER mammalian protein extraction reagent (Pierce, Rockford, IL, USA) containing 1 mM dithiothreitol, 1 mM phenylmethylsulfonyl fluoride, and protease inhibitor cocktail (Roche Diagnostics GmbH, Mannheim, Germany), and subjected to centrifugation at 10,000 rpm for 10 min. Protein concentrations were determined by BCA protein assay. A 30–40 µg aliquot of protein from each treatment was subjected to 10% SDS-PAGE. After electrophoresis, the separated proteins were transferred to Immun-blotTM PVDF membranes (Bio-Rad) by semi-dry blotting, and probed with the appropriate antibody, followed by incubation with Odyssey secondary antibodies, according to manufacturers’ instructions (goat anti-rabbit IRDye 680 or 800 and goat anti-mouse IRDye 680 or 800, depending on required combinations). Blots were imaged and quantitated using an Odyssey Infrared Imaging System (LI-COR Biosciences, Lincoln, NE, USA).

### 4.11. Phospho-Antibody Array Analysis

Phospho-antibody array analysis was performed using a Proteome Profiler Human Phospho-Kinase Array Kit (ARY003B; R&D Systems, Minneapolis, MN, USA). Briefly, MDA-MB-468 cells were treated with either control or 2.0 μM lutein for 12 h. Cells were lysed with Lysis Buffer 6 (R&D Systems, Minneapolis, MN, USA) and agitated for 30 min at 4 °C. Cell lysates were clarified by microcentrifugation at 14,000 × *g* for 5 min and supernatants were assayed for protein concentrations. Preblocked Human Phospho-Kinase Array nitrocellulose membranes were incubated with ∼300 μg of cellular extract overnight at 4 °C on a rocking platform. The membranes were washed three times with 1× Wash Buffer (R&D Systems) to remove unbound proteins, and then incubated with a mixture of biotinylated detection antibodies and streptavidin-HRP antibodies. Chemiluminescent detection reagents were applied to detect spot densities. Array images were analyzed using ImageJ software. Each spot was subtracted by the averaged background level from negative control spots and normalized by the density levels of its own positive control spots to validate results from four different conditions. The averaged density of duplicated spots representing each phosphorylated kinase protein was determined and used for the relative changes in phosphorylated kinase proteins.

### 4.12. Statistical Analysis

Results were expressed as means ± standard derivation (SD) of the number of experiments. Statistical analysis was carried out using a student’s *t*-test or one way Analysis of Variance (ANOVA), followed by Dunnett’s post hoc test, considering *p* < 0.05 to be significant (** *p* < 0.01,* *p* < 0.05).

## 5. Conclusions

We report novel mechanisms by which the carotenoid lutein selectively inhibits growth of breast cancer cells. These intracellular signals involve increased ROS generation, activation of p53 signaling, and increased HSP60 expression. Our findings indicate that lutein may be a non-toxic, selective agent that can induce cell cycle arrest and apoptosis in breast cancer, including triple-negative breast cancer cells. We speculate that the toxicity of taxane chemotherapy might be lessened by enabling lower effective doses in combination with lutein. Further investigations in preclinical and clinical settings should establish lutein as a potential anti-cancer agent in breast cancer.
